# Refractory Dupilumab-Induced Inflammatory Arthritis Treated by Upadacitinib: A ‘Janus Kinase (JAK)’ of All Trades

**DOI:** 10.7759/cureus.68438

**Published:** 2024-09-02

**Authors:** Shiamak Cooper, Ali Mohamed, Anthony Ocon

**Affiliations:** 1 Internal Medicine, Rochester Regional Health, New York, USA; 2 Rheumatology, Rochester Regional Health, New York, USA; 3 Rheumatology, University of Rochester, Rochester, USA

**Keywords:** inflammatory arthritis, atopic dermatitis, drug toxicity, interleukins, biologic therapy

## Abstract

Dupilumab, a monoclonal interleukin (IL)-4 receptor α antagonist, is used to treat moderate-to-severe atopic dermatitis. Uncommonly, inflammatory arthritis and enthesitis may occur upon initiation of dupilumab. Upadacitinib, a Janus kinase (JAK) inhibitor, is an alternative medication approved for moderate-to-severe atopic dermatitis but is also used to treat inflammatory arthritis. We report a case of dupilumab-induced inflammatory arthritis that was refractory to oral nonsteroidal anti-inflammatory drugs (NSAIDs) and corticosteroids and was successfully treated by upadacitinib, which also treated the atopic dermatitis.

A 40-year-old female with moderate-to-severe atopic dermatitis was treated with dupilumab for 10 months, showing improvement in her skin. However, she then developed recurrent right knee effusions, polyarthritis in her hands, feet, and knees, and prolonged stiffness. She noticed swelling which developed abruptly in her right knee, then progressed to multiple joints including fingers, wrists, ankles, and persisted for four weeks prior to seeking additional medical care. She denied any recent preceding trauma. Joint pain was worsened by movement and morning stiffness lasted over two hours. Trials of ibuprofen or celecoxib and application of ice did not alleviate it. She had an elevated erythrocyte sedimentation rate of 29 mm/hr and C-reactive protein of 21.6 mg/dL. She tested negative for antinuclear antibody, rheumatoid factor, anti-cyclic citrullinated protein, human leukocyte antigen B27, Lyme enzyme-linked immunosorbent assay (ELISA), and Western blot. She was initially treated with a prednisone taper, but the symptoms returned upon reaching 10 mg daily. She continued on dupilumab for four weeks, but stopped as the joint symptoms progressed. With cessation, her atopic dermatitis also became active again. Despite stopping the dupilumab, she continued to have diffuse swelling and tenderness in her hands, feet, knees, and wrists over the next 12 weeks. Upadacitinib, within one month of initiating, led to improvement in both joints and skin. She was able to taper off the corticosteroids. At five months, she continued to not have swelling or tenderness in her joints, and her skin was well-controlled.

We report the first successful use of upadacitinib for the treatment of refractory dupilumab-induced inflammatory arthritis as well as atopic dermatitis. The use of JAK inhibitors should be considered to treat this uncommon condition, given that they also treat atopic dermatitis.

## Introduction

Atopic dermatitis is a common inflammatory skin disease related to the underlying dysregulation of T helper 2 (Th2) cells and overexpression of interleukin (IL)-4 [1]. Dupilumab, a monoclonal IL-4 receptor α antagonist, is used to treat moderate-to-severe atopic dermatitis [2]. Uncommonly, inflammatory arthritis and enthesitis may occur upon initiation of dupilumab [3,4,5,6]. Treatment usually includes cessation of dupilumab and nonsteroidal anti-inflammatory drugs (NSAIDs) [7]; however, there is limited guidance on how to treat more refractory dupilumab-induced inflammatory arthritis. Furthermore, atopic dermatitis may worsen upon cessation of dupilumab. Upadacitinib, a Janus kinase (JAK) inhibitor, is an alternative medication approved for moderate-to-severe atopic dermatitis but is also used to treat inflammatory arthritis, such as rheumatoid arthritis, axial spondyloarthritis, and psoriatic arthritis. We report a case of dupilumab-induced inflammatory arthritis that was refractory to oral NSAIDs and corticosteroid medication and was successfully treated by upadacitinib, which also treated the atopic dermatitis.

## Case presentation

A 40-year-old female with moderate-to-severe atopic dermatitis was treated with dupilumab 300 mg subcutaneous injections every two weeks for 10 months, resulting in improvement in her skin. However, she then developed recurrent right knee effusions, polyarthritis in her hands, feet, and knees, and prolonged stiffness. She did not have any enthesitis in her elbow, knee, heel, or plantar fasciitis. She did not have back pain or symptoms suggestive of sacroiliitis, nor did she report dactylitis. However, she noticed swelling which developed abruptly in her right knee, then progressed to multiple joints including fingers, wrists, and ankles, and persisted for four weeks prior to seeking additional medical care. She denied any recent preceding trauma. She had no other past medical history but did have a past surgical history of right knee arthroscopy with anterior cruciate ligament reconstruction over 20 years ago following an injury. Joint pain was worsened by movement. Morning stiffness lasted over two hours, and she experienced a gelling phenomenon with prolonged immobility. Trials of ibuprofen 800 mg every six hours or celecoxib 200 mg twice daily and the application of ice did not alleviate it. She denies a family history of rheumatoid arthritis, psoriatic arthritis, axial spondyloarthritis, other inflammatory joint syndromes, or inflammatory bowel disease. She did have a family history of psoriasis in her grandfather, but she did not have known psoriasis herself. She denied eye redness or pain, abdominal pain, diarrhea, hematochezia or melena, hematuria, other rashes, or adenopathy.

She underwent right knee arthrocentesis as well as an intra-articular 2 ml of triamcinolone 40 mg/ml injection, which was beneficial for only two weeks. Fluid analysis revealed more than 18,000 nucleated cells with 81% polynuclear cells and 19% mononuclear cells, fewer than 3,000 red blood cells, and no crystals. Laboratory assessment found an elevated erythrocyte sedimentation rate of 29 mm/hr (ref range 0-20 mm/hr) and C-reactive protein of 21.6 mg/dL (reference range 0-1 mg/dL). She was negative for antinuclear antibody, rheumatoid factor, anti-cyclic citrullinated protein, human leukocyte antigen B27, Lyme ELISA, and Western blot. MRI of the right knee demonstrated moderate joint effusion, synovitis, minimal patellofemoral degenerative changes, and prior healed meniscal tears.

She was initially treated with a prednisone taper beginning at 20 mg daily and reduced by 5 mg every two weeks, but the symptoms returned upon reaching 10 mg daily. She continued on dupilumab for four weeks, but stopped as the joint symptoms progressed. With cessation, her atopic dermatitis also became active again. Despite stopping the dupilumab, she continued to have diffuse swelling and tenderness of her hands, feet, knees, and wrists over the next 12 weeks. Upadacitinib, known to benefit both atopic dermatitis and inflammatory arthritis, was initiated along with another corticosteroid taper. Within one month of initiating it, she had improvement in both joints and skin. Synovitis in her hand and foot joints resolved. She was able to taper off the corticosteroids. At five months, she continued to not have swelling or tenderness in her joints, and her skin was well-controlled.

## Discussion

We report a case of dupilumab-induced inflammatory arthritis that persisted despite cessation of dupilumab, NSAID therapy, and corticosteroid therapy. The patient responded very well to upadacitinib, showing improvement in both inflammatory arthritis and atopic dermatitis. This is the first report of upadacitinib as a treatment for dupilumab-induced inflammatory arthritis.

Dupilumab-induced inflammatory arthritis and enthesitis is uncommon. The exact incidence is not known but is estimated at around 5% according to a retrospective study of 400 patients [8]. There is minimal literature suggesting how to best manage it, with a prior case report suggesting NSAIDs may be beneficial [7]. However, NSAIDs do not treat atopic dermatitis, and guidance for more refractory cases is not clear. Our case report suggests that upadacitinib should be considered for more severe cases given that it may treat both cutaneous symptoms and inflammatory arthritis.

Pathophysiologically, IL-4/IL-13 receptors occur at enthesis points and the enthesis T cells secrete basal levels of IL-4 [9]. Moreover, IL-4 may decrease inflammation by inhibiting IL-23 in antigen-presenting cells [10]. Thus, inhibition of IL-4 by dupilumab may lead to enthesitis and inflammatory arthritis similar to how it occurs in spondyloarthropathy conditions such as axial spondyloarthritis and psoriatic arthritis. JAK proteins act downstream from various ILs and further activate signal transducer and activator of transcription (STAT) proteins to drive different inflammatory processes. In terms of atopic dermatitis, a predominant Th2 response in the acute phase causes recruitment of IL-4, 5, 13, and 31, which promotes local inflammation. These cytokines bind to their respective receptors and exert their effects via JAK-STAT signal transduction [11], causing downregulation of filaggrin gene expression and skin barrier dysfunction, playing a role in atopic dermatitis [12]. A key role in inflammatory arthritis is played by Th17 cells and IL-17 [13]. IL-17 receptor expression is thought to be increased in synovial fibroblasts of patients with rheumatoid arthritis (RA) and psoriatic arthritis (PsA) [14]. In addition, JAK continues to be present in the downstream pathway to exert the effects of IL-17 [15]. Thus, JAK inhibition likely prevents the activity of IL-4/IL-13 involved in the inflammation of atopic dermatitis as well as treats the inflammatory arthritis due to its effect on curbing the IL-23/17 pathway.

## Conclusions

Dupilumab may cause a rare inflammatory arthritis and enthesitis. We report the first successful use of upadacitinib for the treatment of refractory dupilumab-induced inflammatory arthritis, as well as atopic dermatitis. The use of JAK inhibitors should be considered to treat this uncommon condition given their effectiveness in treating atopic dermatitis. Prospective clinical trials are necessary to confirm these findings.
